# *Staphylococcus aureus* susceptibilities from wounds of patients who use illicit fentanyl

**DOI:** 10.1017/ash.2025.12

**Published:** 2025-02-12

**Authors:** Drew T. Dickinson, Stephen Saw, Lauren Dutcher, Christina Maguire, Adrienne Terico, Margaret Lowenstein, Sonal Patel

**Affiliations:** 1 Department of Pharmacy Services, Hospital of the University of Pennsylvania, Philadelphia, PA, USA; 2 Division of Infectious Diseases, Department of Medicine, University of Pennsylvania Perelman School of Medicine, Philadelphia, PA, USA; 3 Department of Pharmacy Services, Penn Presbyterian Medical Center, Philadelphia, PA, USA; 4 Department of Pharmacy Services, Pennsylvania Hospital, Philadelphia, PA, USA; 5 Center for Addiction Medicine & Policy, University of Pennsylvania, Philadelphia, PA, USA; 6 Division of General Internal Medicine, Department of Medicine, University of Pennsylvania Perelman School of Medicine, Philadelphia, PA, USA

## Abstract

**Objective:**

Develop a *Staphylococcus aureus* wound antibiogram among patients who use fentanyl (PWUF) presenting with acute *S. aureus* skin and soft tissue infections (SSTIs) in Philadelphia, Pennsylvania.

**Design:**

Retrospective, multisite cohort study.

**Patients and Setting:**

Individuals presenting to emergency departments or admitted to inpatient units of four Penn Medicine hospitals with an acute *S. aureus* SSTI and illicit fentanyl use within the previous year.

**Methods:**

We described susceptibilities of *S. aureus* isolated from wound cultures among the PWUF cohort and compared these to the health system’s wound antibiogram. We compared frequency of in-hospital medication treatment for opioid use disorder among patients who left the hospital prior to vs after the availability of *S. aureus* susceptibilities.

**Results:**

Among 131 *S. aureus* isolates from 131 PWUF, 35/131 (26.7%) were susceptible to oxacillin, 73/121 (60.3%) were susceptible to clindamycin, 77/122 (63.1%) were susceptible to tetracycline, and 119/126 (94.4%) were susceptible to trimethoprim-sulfamethoxazole. PWUF displayed significantly reduced susceptibility to oxacillin and tetracycline compared to the health system’s outpatient wound *S. aureus* antibiogram. Compared to patients discharged prior to susceptibility availability, more patients discharged after the reporting of susceptibilities were administered buprenorphine or methadone in the hospital (82.0% vs 51.4%, *P* < 0.001).

**Conclusion:**

High nonsusceptibility to clindamycin and tetracycline suggests these agents should not be prescribed as empiric therapy for acute *S. aureus* SSTI in PWUF in Philadelphia. PWUF would benefit from joint management by infectious diseases and addiction medicine experts to ensure prescription of active therapy. Additional study is needed of PWUF in other regions.

## Introduction

*Staphylococcus aureus* is commonly isolated from wounds of people who use drugs (PWUD).^1–3^ Risk factors such as breach of skin barriers, shared injection equipment, and insecure housing place these individuals at high risk for skin and soft tissue infections (SSTIs).^4–8^

Isolates of *S. aureus* among PWUD have displayed varied resistance patterns with high prevalence of resistance to common oral agents used to treat methicillin-resistant *S. aureus* (MRSA).^1,9^ Provision of appropriate antibiotic therapy in the setting of a suspected *S. aureus* SSTI can be complicated as PWUD may leave the hospital before medically advised (BMA), also known as self-directed discharge, often in the setting of incompletely treated withdrawal.^5,10,11^ Additionally, patients presenting to the emergency department who do not require hospital admission may be discharged before complete microbiologic data is available. When discharged before the return of culture results and susceptibilities, inactive antibiotic therapy may be inadvertently empirically prescribed upon discharge, placing patients at risk for poor outcomes.^12^

In Philadelphia, like most of the rest of the US, fentanyl has become the leading substance implicated in unintentional overdose deaths.^13^ As MRSA prevalence among PWUD has also increased,^14^ there has been little data describing recent susceptibility patterns among this group to help guide empiric treatment. This is especially salient as fentanyl use is associated with frequent injections and ulcerogenic substances, such as xylazine, have infiltrated the drug supply.^15,16^ We conducted a multisite retrospective cohort study of patients who use illicit fentanyl (PWUF) presenting with *S. aureus* SSTI to describe susceptibility patterns among *S. aureus* isolates, particularly for commonly used oral agents that might be prescribed for patients discharged early. We also identified factors associated with prescription of active antibiotic therapy upon discharge and risk factors for *S. aureus* resistance.

## Methods

We conducted a multisite, retrospective cohort study across four hospitals in Philadelphia, Pennsylvania of PWUF presenting with acute *S. aureus* SSTI (Table 1).

**Table 1.** Characteristics of included hospitals

	Hospital A	Hospital B	Hospital C	Hospital D
Inpatient Bed Size	1,094	399	525	106
Annual Inpatient Admissions	33,871	17,314	19,310	4,740
Annual OUD^a^-Related Admissions	1020	844	654	533
On-Site Addiction Medicine Consult Service	No	Yes	No	No
On-Site Psychiatric Consult Service	Yes	Yes	Yes	Yes
On-Site Infectious Diseases Consult Service	Yes	Yes	Yes	Yes
Infectious Diseases Stewardship Pharmacist FTE^b^	3.0	2.0	1.0	0
Opioid Stewardship Pharmacist FTE^b^	1.0	0	0	0

a Opioid use disorder: defined by ICD-10 codes for opioid use and overdose during hospitalization (F11.1, F11.2, F11.9, T40).

b Full-time equivalent.

### Ethics

Research was conducted in accordance with the Declaration of Helsinki. This study, including a waiver of informed consent, was approved by the Institutional Review Board of the University of Pennsylvania (Protocol Number: 833186).

### Study period and participants

Adult patients who presented to the emergency department or who were hospitalized in an inpatient unit with *S. aureus* isolated from a skin or soft tissue culture between July 1, 2021, and June 30, 2023, were included. In the instance *S. aureus* was isolated from multiple eligible cultures from an individual during the study period, only the first culture per patient was included. Bedside as well as operating room cultures were included. We chose to include patients using fentanyl given the predominance of fentanyl use in Philadelphia and fentanyl use could be confirmed through urine drug screen (UDS). Patients must have had a UDS positive for fentanyl or its metabolite (norfentanyl) within 1 year prior to collection of the index culture. Patients must have had documentation of active substance use disorder in the electronic health record (EHR). Documentation was not limited to injection drug use given the multiple modalities of fentanyl consumption (ie, injection or inhalation). To characterize *S. aureus* isolates associated with fentanyl use as opposed to nosocomial acquisition, index cultures collected more than 48 hours after presentation were excluded.

### Outcomes (dependent variables)

The primary outcome was description of *S. aureus* susceptibilities to develop an antibiogram of *S. aureus* isolated from skin and soft tissue cultures among PWUF. Susceptibilities of *S. aureus* were abstracted from the finalized susceptibility report. Isolates reported as intermediate in the finalized report were classified as nonsusceptible. If multiple strains of *S. aureus* were isolated from the index skin and soft tissue culture, the susceptibility profile of the more resistant strain was collected. Susceptibility to tetracycline was used as a surrogate for doxycycline susceptibility.

Secondary outcomes included length of stay, discharge disposition, prescription of active therapy upon discharge, 30-day hospital representation, administration of medication for opioid use disorder, and maximum clinical opiate withdrawal scale (COWS) score.^17^ Active therapy upon discharge was defined as prescription of an antibiotic that had demonstrated *in vitro* susceptibility. Patients who completed antibiotic therapy in the hospital were excluded from active therapy analysis; however, patients discharged BMA without prescription of antibiotics were classified as being discharged on inactive therapy. We compared the time at which *S. aureus* identification and susceptibility results populated in the EHR to the time of discharge to quantify the number of patients who discharged prior to *S. aureus* identification and susceptibility. Lastly, risk factors for nonsusceptibility to oxacillin, clindamycin, tetracycline, and trimethoprim-sulfamethoxazole were identified in an exploratory analysis. Risk factors for these agents were identified given their importance as oral therapy options for treatment of *S. aureus* infections.

### Exposures (independent variables) and data sources

Data were abstracted from the EHR (Hyperspace 2023; Epic Systems Corporation). Baseline demographics included age, sex, race, ethnicity, and past medical history. Housing status was abstracted from social work evaluation. Microbiologic data during and 90 days prior to the index hospitalization were collected. Data regarding fentanyl use included qualifying UDS, administration of buprenorphine or methadone during index presentation, and maximum COWS score during index presentation. Healthcare utilization included hospital admission, intensive care unit transfer, length of stay, antibiotic utilization, hospital presentation 90 days prior to and 30 days following the index visit, and discharge disposition.

### Statistical analysis

Data were collected and stored using a centralized database (REDCap, West Lafayette, IN).^18,19^ Baseline demographics, substance use, and healthcare utilization data were summarized descriptively. Proportions were reported for categorical variables and medians and interquartile ranges (IQR) were reported for continuous variables. Susceptibilities of *S. aureus* to an antibiotic were calculated as a percentage of all isolates for which a susceptibility was tested and reported. We compared susceptibilities of *S. aureus* among PWUF to the health system’s outpatient December 2021 to December 2022 antibiogram for wound cultures using the chi-square test. The health system’s outpatient antibiogram was selected as this antibiogram contains patients with community-onset infections as well as patients who present to the emergency department, most closely resembling the PWUF cohort. Patients discharged prior to the availability of *S. aureus in vitro* susceptibilities were compared to patients discharged after the availability of susceptibilities using the chi-square or Mann–Whitney U test as appropriate. A binomial multivariable regression model was developed to identify risk factors for nonsusceptibility to oxacillin, clindamycin, tetracycline, and trimethoprim-sulfamethoxazole, controlling for housing, healthcare exposure within previous 90 days, and IV antibiotic use within previous 90 days. Exposure to first-generation cephalosporins, clindamycin, doxycycline, and trimethoprim sulfamethoxazole within previous 90 days were also tested for model inclusion. All p-values were two-sided, and a p-value of <0.05 was considered statistically significant. Analyses were conducted in SAS version 9.4 (Cary, NC).

## Results

**Figure 1.**
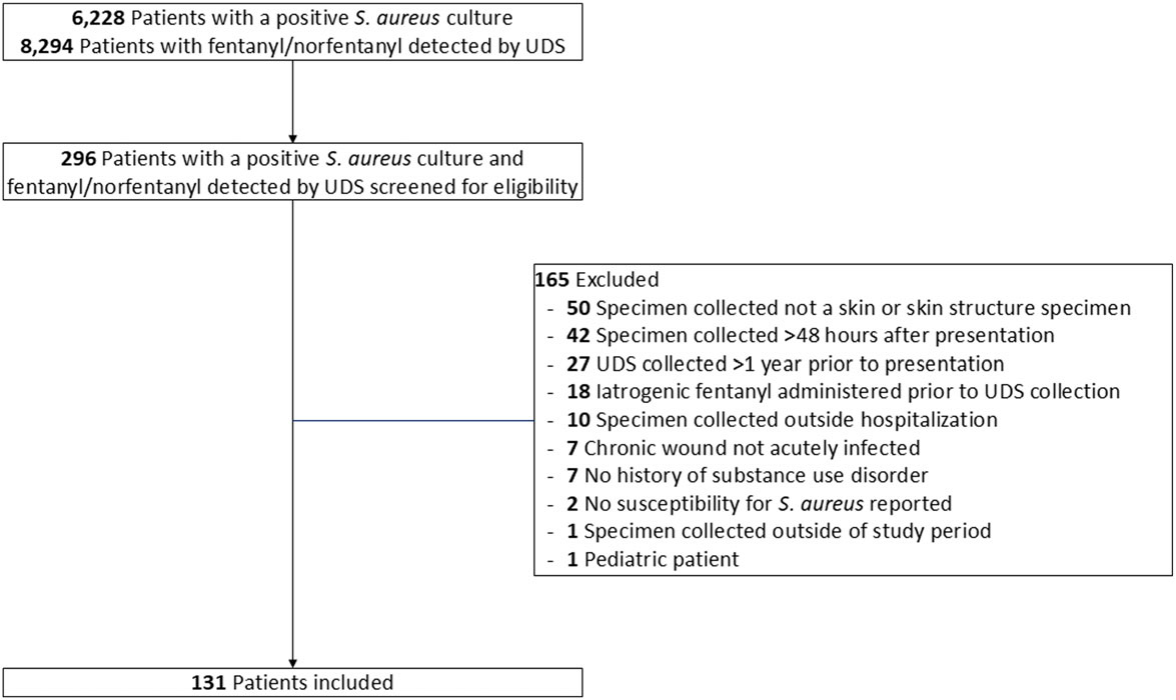
Patients presenting with acute *S. aureus* SSTI with documented fentanyl use within the previous year.

Between July 1, 2021, and June 30, 2023, 131 adult patients presenting with a community-onset acute *S. aureus* SSTI with fentanyl use within the preceding year were included (Figure 1). The median age of the cohort was 37 (IQR, 33–43) years (Table 2). Patients were predominantly male (80 patients, 61.1%) and white (99 patients, 78.6%). Fifty-one (47.7%) patients were identified as unhoused.

**Table 2.** Demographics and healthcare utilization of PWUF with acute *S. aureus* SSTI

	Total (N = 131) n (%)
**Demographics and Clinical Characteristics**	
Age (years)^†^	37 (33-43)
Male	80 (61.1%)
Race (n = 126)	
White	99 (78.6%)
Black	19 (15.1%)
Other	8 (6.3%)
Ethnicity (n = 129)	
Hispanic	10 (7.8%)
Non-Hispanic	119 (92.2%)
Unhoused (n = 107)	51 (47.7%)
Diabetes	4 (3.1%)
Immunocompromised^‡^	2 (1.5%)
Positive MRSA^a^ Nasal Swab (n = 49)	24 (49.0%)
Concomitant *S. aureus* Bacteremia	9 (6.9%)
**Healthcare Utilization**	
Hospital Admission	110 (84.0%)
ICU^b^ Admission	8 (6.1%)
Length of Stay (days)^†^	2.9 (1.5-6.9)
Discharge Before *S. aureus* Identification	43 (32.8%)
Discharged Before *S. aureus S*usceptibility Report	70 (53.4%)
Discharge Before Medically Advised (n = 130)	60 (46.2%)
Hospital or ED^c^ Presentation in Previous 90 Days	87 (66.4%)
Representation to Hospital Within 30 Days of Discharge	55 (42.0%)

† Presented as median (interquartile range).

‡ Patients were classified as immunocompromised if they met any of the following: absolute neutrophil count less than 1,000 neutrophils/mm^3^ within 48 hours of presentation, diagnosis of a hematologic malignancy, receipt of bone marrow transplant, receipt of chemotherapy within the previous 30 days, receipt of solid organ transplant, or HIV infection with a CD4+ count less than 50 cells/mm^3^ within 30 days prior to presentation.

a MRSA, methicillin-resistant *S. aureus.*

b ICU, intensive care unit.

c ED, emergency department.

Most patients required inpatient admission. Among all patients, 43 (32.8%) patients were discharged prior to the identification of *S. aureus* from culture and 70 (53.4%) patients were discharged prior to availability of *S. aureus* susceptibilities. Nearly one-half of patients were discharged BMA (60 patients, 46.2%). Among these patients, 27 (45.0%) patients were discharged prior to the identification of *S. aureus* from culture and 46 (76.7%) patients were discharged prior to the availability of *S. aureus* susceptibilities. Most patients were discharged from an internal medicine (91 patients, 69.5%) or infectious diseases (21 patients, 16.0%) service.

Collective susceptibilities of *S. aureus* from skin and soft tissue cultures of PWUF are displayed in Table 3. The majority of isolates were MRSA with 96 (73.3%) isolates nonsusceptible to oxacillin. There was a high degree of resistance to oral agents commonly used to treat *S. aureus* SSTIs. Over one-third of isolates were nonsusceptible to clindamycin, and over one-third of isolates were nonsusceptible to tetracycline. Among 117 isolates with susceptibilities reported for clindamycin, tetracycline, and trimethoprim-sulfamethoxazole, 77 (65.8%) isolates were nonsusceptible to at least one of these agents and 19 (16.2%) isolates were nonsusceptible to two agents. No isolate was nonsusceptible to all three agents. Compared to the health system’s outpatient wound antibiogram, *S. aureus* isolated from PWUF displayed significantly greater oxacillin and tetracycline nonsusceptibility (Table 3). All isolates were susceptible to vancomycin.

**Table 3.** Antibiogram comparing susceptible *S. aureus* isolates from the wounds of PWUF to *S. aureus* isolates from the wounds of the health system’s total outpatient population

	PWUF^a^ Wound Cultures		Health System Outpatient Wound Cultures	P-value
Clindamycin	73/121 (60.3%)		595/862 (69.0%)	0.0549
	MSSA^b^ 17/28 (60.7%)	MRSA^c^ 56/93 (60.2%)		
Oxacillin	35/131 (26.7%)		641/937 (68.4%)	<0.0001
Tetracycline	77/122 (63.1%)		749/870 (86.1%)	<0.0001
	MSSA 25/29 (86.2%)	MRSA 52/93 (55.9%)		
Trimethoprim-Sulfamethoxazole	119/126 (94.4%)		753/778 (96.8%)	0.187
	MSSA 34/35 (97.1%)	MRSA 85/91 (93.4%)		

a PWUF: persons who use fentanyl.

b MSSA, methicillin-susceptible *S. aureus.*

c MRSA: methicillin-resistant *S. aureus.*

Patients received a median of 3 (IQR, 2–8) days of inpatient *S. aureus* antibiotic therapy. Those who were discharged on antibiotics received a median of 10 (IQR, 6–10) days of antibiotics on discharge. Among patients who discharged BMA, 30 (50.0%) patients were discharged without an antibiotic prescription. Antibiotics administered in the hospital and prescribed upon discharge can be found in Supplemental Table 1.

Seventy (53.4%) patients were discharged prior to the availability of *S. aureus* susceptibilities (Table 4). Fewer patients discharged after susceptibility availability were discharged BMA compared to patients discharged before susceptibility availability (14/61 (23.0%) patients vs 46/69 (66.7%) patients; *P* < 0.001). Excluding eight patients who completed antibiotic therapy in the hospital, 41 (80.4%) patients discharged after susceptibility availability were discharged on active therapy compared to 33 (49.3%) patients discharged before susceptibility availability (*P* < 0.001). A greater proportion of patients discharged after susceptibility availability were administered methadone or buprenorphine during their hospitalization compared to patients with earlier discharges (50 (82.0%) patients vs 36 (51.4%) patients; *P* < 0.001).

**Table 4. tbl4:** Comparison of patients discharged before and after *S. aureus* susceptibility availability

	Discharged Before Susceptibilities (n = 70)	Discharged After Susceptibilities (n = 61)	P-value
Length of Stay (days)^†^	1.5 (0.7–2.1)	7.3 (4.9–13.3)	<0.0001
Discharged on Active Therapy	33/67 (49.3%)	41/51 (80.4%)	0.0005
Discharged BMA^a^	46/69 (66.7%)	14 (23.0%)	<0.0001
Administered Buprenorphine or Methadone	36 (51.4%)	50 (82.0%)	0.0002
Maximum COWS^b^ Score^†^	10 (4.0-14.0)	11 (7.5-13.0)	0.2816
30-Day Re-presentation	29 (41.4%)	26 (42.6%)	0.8901

† Presented as median (interquartile range).

a BMA: before medically advised.

b COWS: clinical opiate withdrawal scale.

In 31 patients with an MSSA infection, 26 (83.9%) were prescribed active therapy on discharge based on *in vitro* susceptibilities. In 87 patients with an MRSA infection, 48 (55.2%) were prescribed active therapy on discharge. Among 10 patients prescribed clindamycin on discharge, 2 (20%) isolates demonstrated *in vitro* nonsusceptibility. Among 35 patients prescribed doxycycline on discharge, 9 (25.7%) demonstrated *in vitro* nonsusceptibility to tetracycline. All 26 patients prescribed trimethoprim-sulfamethoxazole on discharge received active therapy.

In the exploratory analysis, significant risk factors for antibiotic nonsusceptibility were only identified for tetracycline (Table 5). In bivariate analysis, hospitalization in the previous 90 days (risk ratio [RR] 3.054, P = 0.005) and receipt of intravenous antibiotics within the previous 90 days (RR 2.346, P < 0.001) were significant predictors of tetracycline nonsusceptibility. These parameters remained significant predictors of increased risk of tetracycline nonsusceptibility in multivariable analysis. No significant predictors of oxacillin, clindamycin, or trimethoprim-sulfamethoxazole nonsusceptibility were identified (Supplemental Table 3).

**Table 5.** Exploratory analysis for risk factors for tetracycline nonsusceptibility among *S. aureus* isolated from persons who use fentanyl

	Bivariate Analysis		Multivariable Analysis	
	RR^a^ (95% CI^b^)	P-value	aRR^c^ (95% CI)	P-value
Unhoused	1.370 (0.838 – 2.239)	0.210	1.306 (0.868 – 1.962)	0.201
Hospitalization in Previous 90 Days	3.054 (1.413 – 6.601)	0.005	3.398 (1.272 -9.079)	0.015
Intravenous Antibiotic Exposure in Previous 90 Days	2.346 (1.548 – 3.555)	<0.001	1.760 (1.120 – 2.766)	0.014
Doxycycline Exposure in Previous 90 Days	1.668 (0.988 – 2.815)	0.055	1.006 (0.656 – 1.544)	0.978

a RR: risk ratio.

b CI: confidence interval.

c aRR: adjusted risk ratio.

## Discussion

In our analysis of *S. aureus* wound isolates in PWUF in Philadelphia, we found significantly greater nonsusceptibility to oxacillin, clindamycin, and tetracycline compared to isolates from our health system’s general outpatient population. Of the three oral agents routinely prescribed for MRSA infections, doxycycline was the most commonly prescribed discharge antibiotic in our study. Doxycycline demonstrates high *in vitro* activity against *S. aureus* among general outpatients in our health system’s antibiogram, making it an often-preferred antibiotic for empiric and targeted *S. aureus* therapy, in addition to its standardized dosing and high tolerability compared to clindamycin and trimethoprim-sulfamethoxazole. However, one-quarter of PWUF discharged with doxycycline were presumably discharged on inactive therapy. Lloyd-Smith and colleagues analyzed susceptibility patterns among 37 *S. aureus* isolates from the wounds of patients presenting to a Vancouver supervised injection facility in 2008. Roughly half of *S. aureus* isolates were MRSA and 92% of all isolates demonstrated susceptibility to tetracycline.^1^ Differences in resistance profiles between that study and ours may, in part, be attributed to the changing epidemiology of *S. aureus* over time. Tetracycline resistance has significantly increased over the past decade among outpatients with MRSA infections.^20^ The USA300 clone has become the predominant clone implicated in SSTIs within the United States.^21,22^ USA300 has acquired methicillin, clindamycin, and tetracycline resistance and has been theorized to move westward across North America within the past 25 years, possibly accounting for the difference in susceptibilities from Lloyd-Smith and colleagues.^23–25^ Non-USA300 clones, including sequence types 5 and 9, may also contribute to the high-degree of drug resistance, including tetracyclines.^26,27^ Additionally, *S. aureus* transmission has been demonstrated to be dependent on drug-use networks.^28,29^ Infections among social networks of PWUF within Philadelphia may be enriched with tetracycline-resistant *S. aureus* compared to the general population or PWUF in other geographic regions.

Roughly half of patients were discharged prior to the availability of *S. aureus* susceptibilities. Hazen and colleagues estimated that 15% of patients with SSTIs secondary to injection drug use directed their own discharge from the hospital, placing these patients at increased risk for 90-day readmission.^30^ Managing withdrawal by administering medications for opioid use disorder to patients with serious infections to prevent premature discharge have been met with mixed success.^10,31^ Within our study, patients discharged prior to susceptibility availability were more likely to be discharged BMA. These patients were also less frequently administered methadone or buprenorphine compared to patients discharged after susceptibilities were available. Methods of encouraging patients to stay hospitalized until more microbiologic data become available are crucial to ensure active oral therapy upon discharge. For those that do elect to leave the hospital, advances in rapid molecular diagnostics for *S. aureus* susceptibilities as well as multidisciplinary follow-up can limit discharges on inactive therapy.^32^ This highlights the need for joint infectious diseases and addiction medicine management of patients who use drugs presenting with acute SSTI. Opioid withdrawal is often difficult to control as the potency of illicit opioids has increased and opioids are frequently adulterated with other substances, such as xylazine, that may complicate withdrawal.^33,34^ In the face of an evolving antimicrobial resistance and drug-use landscape, further research across disciplines is needed to better provide antimicrobial therapy for patients at risk for opioid withdrawal.

Transitions of care for PWUF with a *S. aureus* infection are difficult. With the high incidence of resistance to commonly used oral *S. aureus* agents, prescription of active empiric therapy on discharge is challenging. A study of patients who inject drugs that were prescribed oral antibiotic therapy after discharging BMA estimated that nearly one-quarter of patients were lost to follow-up after discharge.^32^ Thus, these patients may be difficult to contact after discharge to inform of the need to switch antibiotic therapy to an active *in vitro* agent. Despite recent evidence, PWUF are often not considered candidates for outpatient parenteral antimicrobial therapy.^35^ Prescription of oral linezolid on discharge carries a risk of serotonin syndrome when combined with fentanyl use. While the risk of serotonin syndrome with medically prescribed doses of fentanyl is low, the risk with substantially higher illicit doses requires further investigation.^36^ Highly active oral anti-MRSA agents, such as delafloxacin, omadacycline, and tedizolid, are cost-prohibitive for many PWUF without commercial insurance. For those with medical coverage, these agents often require prior authorization. Patients may be unwilling to stay hospitalized during benefits investigation, particularly when experiencing opioid withdrawal symptoms. Half of patients discharged BMA were discharged without an antibiotic. Patients may elect to leave the hospital quickly, leaving little time to prescribe antibiotics to an outpatient pharmacy. The inpatient use of long-acting lipoglycopeptides, such as dalbavancin and oritavancin, are attractive options to provide adequate therapy for SSTIs administered as a single dose. While the high cost of lipoglycopeptides has come under scrutiny by health systems, prevention of treatment failure and subsequent readmission may offset the cost.^37^

Our study has several limitations. Primarily, isolation of *S. aureus* could represent superficial colonization as opposed to active infection. We restricted inclusion to patients who were determined to be acutely infected and required antibiotic therapy per the treating provider. However, *S. aureus* may not have been an infecting pathogen among some PWUF given the polymicrobial nature of many cultures and nonsterile collection. Second, i*n vitro* susceptibility testing for tetracycline at our institution is used as a surrogate for doxycycline. Isolates may have maintained susceptibility to doxycycline, inflating estimates of doxycycline nonsusceptibility and the proportion of patients discharged on inactive doxycycline therapy. Third, we were unable to obtain antibiotic administration data outside of our health system. This population is highly transient with frequent healthcare presentations; therefore, measures of previous antibiotic exposure are underestimated.^38^ Associations between *S. aureus* susceptibility and prior antibiotic exposure were likely underpowered. Fourth, pathogens other than *S. aureus* present in wound cultures were not collected from the EHR. We were unable to estimate the burden of polymicrobial infections, and antibiotics prescribed may have had indications other than *S. aureus* infection. Lastly, mode of fentanyl use was not collected. As such, *S. aureus* risk and susceptibility as a factor of route of drug use was not assessed. However, it is assumed that most fentanyl use in Philadelphia is via injection.

To our knowledge, this is the largest analysis of *S. aureus* susceptibility among PWUF within a large, multisite hospital network. Our results suggest that clindamycin and doxycycline should not be prescribed empirically in this population for acute *S. aureus* SSTI, particularly within Philadelphia, given high rates of *in vitro* nonsusceptibility. PWUF are medically complex. Highly resistant, often polymicrobial infections, and complex substance withdrawal necessitate collaboration with experts in infectious diseases, emergency medicine, psychiatry, and addiction medicine to provide optimal care. Institutional protocols that provide joint guidance on controlling opioid withdrawal and optimal antibiotic therapy based upon local susceptibility patterns warrant development.

## Supporting information

Dickinson et al. supplementary material Dickinson et al. supplementary material

## Supplementary material

For supplementary material accompanying this paper visit http://doi.org/10.1017/ash.2025.12. click here to view supplementary material
