# Supplementary material for: Staphylococcus aureus susceptibilities from wounds of patients who use illicit fentanyl
Source: Antimicrob Steward Healthc Epidemiol. 2025 Feb 12;5(1):e43. doi: 10.1017/ash.2025.12 (PMC11822584; doi:10.1017/ash.2025.12)
Supplement: Dickinson et al. supplementary material [file S2732494X25000129sup001.docx]

**Supplemental Table 1a**. Hospital administered antibiotics with presumed *in vitro* *S. aureus* activity.

|  | All Patients (N=131) | | Patients with MRSA (n=96) | | Patients with MSSA (n=35) | |
| --- | --- | --- | --- | --- | --- | --- |
|  | n (%)^†^ | Median Duration (days) (IQR) | n (%)^†^ | Median Duration (days) (IQR) | n (%)^†^ | Median Duration (days) (IQR) |
| Vancomycin (Intravenous) | 112 (85.5%) | 3 (2-4) | 82 (85.4%) | 3 (2-4) | 30 (85.7%) | 2.5 (2-4) |
| Cefepime | 39 (29.8%) | 3 (1-4) | 28 (29.2%) | 3 (1-4) | 11 (31.4%) | 2 (1-4) |
| Piperacillin-tazobactam | 41 (31.3%) | 2 (1-3) | 30 (31.3%) | 2.5 (2-4) | 11 (31.4%) | 2 (1-3) |
| Doxycycline | 26 (19.9%) | 2 (1-3) | 22 (22.9%) | 2 (1-3) | 4 (11.4%) | 1.5 (1-2.5) |
| Trimethoprim-sulfamethoxazole | 24 (18.3%) | 2 (1-4) | 19 (19.8%) | 3 (1-4) | 5 (14.3%) | 1 (1-2) |
| Ceftriaxone | 22 (16.8%) | 2 (1-3) | 16 (16.7%) | 1.5 (1-3) | 6 (17.1%) | 2 (1-3) |
| Clindamycin | 22 (16.8%) | 1.5 (1-3) | 15 (15.6%) | 2 (1-4) | 7 (20.0%) | 1 (1-3) |
| Cefazolin | 16 (12.2%) | 3 (1.5-7) | 7 (7.3%) | 1 (1-3) | 9 (25.7%) | 5 (3-8) |
| Amoxicillin-clavulanate | 12 (9.2%) | 2.5 (1-7.5) | 9 (9.4%) | 2 (1-4) | 3 (8.7%) | 6 (3-9) |
| Ampicillin-sulbactam | 10 (7.6%) | 3 (2-5) | 5 (5.2%) | 2 (2-3) | 5 (14.3%) | 4 (3-5) |
| Cefadroxil | 10 (7.6%) | 5 (3-6) | 4 (4.2%) | 3 (2.5-4) | 6 (17.1%) | 5.5 (5-6) |
| Levofloxacin | 6 (4.6%) | 6.5 (1-10) | 4 (4.2%) | 2 (1-6.5) | 2 (5.7%) | 11.5 (10-13) |
| Cephalexin | 5 (3.8%) | 1 (1-1) | 3 (3.1%) | 1 (1-1) | 2 (5.7%) | 4 (1-7) |
| Daptomycin | 3 (2.3%) | 5 (1-16) | 3 (3.1%) | 5 (1-16) | 0 | - |
| Ertapenem | 1 (0.8%) | 7 | 0 | - | 1 (2.9%) | 7 |
| Meropenem | 1 (0.8%) | 1 | 0 | - | 1 (2.9%) | 1 |
| None | 1 (0.8%) | - | 0 | - | 1 (2.9%) | - |

IQR: interquartile range; MRSA: methicillin-resistant *S. aureus*; MSSA: methicillin-susceptible *S. aureus*

^†^ Sum of patients may be greater than total as patients may have received multiple antibiotics with *S. aureus* activity.

**Supplemental Table 1b.** Antibiotics with presumed *in vitro* *S. aureus* activity prescribed on hospital discharge.

|  | All Patients (N=131) | | Patients with MRSA (n=96) | | Patients with MSSA (n=35) | |
| --- | --- | --- | --- | --- | --- | --- |
|  | n (%)^†^ | Median Duration (days) (IQR) | n (%)^†^ | Median Duration (days) (IQR) | n (%)^†^ | Median Duration (days) (IQR) |
| Doxycycline | 37 (28.2%) | 10 (5-10) | 30 (31.3%) | 8.5 (4-10) | 7 (20.0%) | 10 (7-10) |
| Trimethoprim-sulfamethoxazole | 26 (19.8%) | 10 (6-12) | 21 (21.9%) | 10 (6-12) | 5 (14.3%) | 10 (7-10) |
| Amoxicillin-clavulanate | 18 (13.7%) | 10 (4-10) | 12 (12.5%) | 8.5 (4-10) | 6 (17.1%) | 10 (8-10) |
| Cefadroxil | 14 (10.7%) | 6.5 (4-10) | 6 (6.3%) | 5 (4-7) | 8 (22.9%) | 8.5 (5-10.5) |
| Clindamycin | 10 (7.6%) | 10 (7-10) | 7 (7.3%) | 8 (7-10) | 3 (8.6%) | 10 (10-28) |
| Levofloxacin | 8 (6.1%) | 7.5 (5-28) | 4 (4.2%) | 5 (3.5-7) | 4 (11.4%) | 28 (17-29) |
| Cephalexin | 5 (3.8%) | 10 (5-10) | 2 (2.1%) | 10 (10-10) | 3 (8.6%) | 5 (4-10) |
| Tedizolid | 2 (1.5%) | 29 (38-30) | 1 (1.0%) | 28 | 1 (2.9%) | 30 |
| Cefazolin | 1 (0.8%) | 29 | 0 | - | 1 (2.9%) | 29 |
| Linezolid | 1 (0.8%) | 14 | 1 (1.0%) | 14 | 0 | - |
| Vancomycin (Intravenous) | 1 (0.8%) | 25 | 1 (1.0%) | 25 | 0 | - |
| None | 38 (29.0%) | - | 31 (32.3%) | - | 7 (20.0%) | - |
| Patient-directed discharge | 30 (22.9%) | - | 26 (27.1%) | - | 4 (11.4%) | - |
| Non-patient-directed discharge | 8 (6.1%) | - | 5 (5.2%) | - | 3 (8.6%) | - |

IQR: interquartile range

^†^ Sum of patients may be greater than 131 as patients may have received multiple antibiotics with *S. aureus* activity.

**Supplemental Table 2.** Patients discharged on inactive therapy.

|  | **Patietns discharged on therapy that demonstrated *in vitro* resistance, n (%)** |
| --- | --- |
| ***S. aureus*** |  |
| MSSA | 5/31 (25.8%) |
| MRSA | 39/87 (44.8%) |
| **Discharge antibiotic** |  |
| Clindamycin | 2/10 (20%)   - 2/4 (50%) among those discharged prior to return of susceptibilities |
| Doxycycline | 9/35 (25.71%)   - 6/18 (33.33%) among those discharged prior to return of susceptibilities |
| TMP-SMX | 0/26 (0%)   - 0/13 (0%) among those discharged prior to return of susceptibilities |

MRSA: methicillin-resistant *S. aureus*; MSSA: methicillin-susceptible *S. aureus*

**Supplemental Table 3a**. Exploratory analysis for risk factors for oxacillin non-susceptibility among *S. aureus* isolated from persons who use fentanyl.

|  | Bivariate Analysis | | Multivariable Analysis | |
| --- | --- | --- | --- | --- |
|  | RR (95% CI) | p-value | aRR (95% CI) | p-value |
| Unhoused | 1.043 (0.828-1.314) | 0.720 | 1.057 | 0.618 |
| Hospitalization in previous 90 days | 1.228 (0.957-1.576) | 0.106 | 1.352 | 0.058 |
| Intravenous antibiotic exposure in previous 90 Days | 1.214 (0.993-1.484) | 0.059 | 1.058 | 0.647 |
| First-generation cephalosporin exposure in previous 90 days | 1.129 (0.836-1.523) | 0.429 | - | - |

aRR: adjusted risk ratio; CI: confidence interval; RR: risk ratio

**Supplemental Table 3b**. Exploratory analysis for risk factors for clindamycin non-susceptibility among *S. aureus* isolated from persons who use fentanyl.

|  | Bivariate Analysis | | Multivariable Analysis | |
| --- | --- | --- | --- | --- |
|  | RR (95% CI) | p-value | aRR (95% CI) | p-value |
| Homelessness | 0.950 (0.574-1.573) | 0.843 | 0.966 (0.583-1.599) | 0.892 |
| Hospitalization in previous 90 days | 1.281 (0.769-2.132) | 0.342 | 1.158 (0.630-2.131) | 0.637 |
| Intravenous antibiotic exposure in previous 90 Days | 1.161 (0.708-1.901) | 0.555 | 1.122 (0.617-2.040) | 0.707 |
| Clindamycin exposure in previous 90 days | No convergence | - | No convergence | - |

aRR: adjusted risk ratio; CI: confidence interval; RR: risk ratio

**Supplemental Table 3c**. Exploratory analysis for risk factors for trimethoprim-sulfamethoxazole non-susceptibility among *S. aureus* isolated from persons who use fentanyl.

|  | Bivariate Analysis | | Multivariable Analysis | |
| --- | --- | --- | --- | --- |
|  | RR (95% CI) | p-value | aRR (95% CI) | p-value |
| Homelessness | 1.469 (0.346-6.240) | 0.602 | 1.552 (0.359-6.709) | 0.557 |
| Hospitalization in previous 90 days | 1.250 (0.253-6.176) | 0.784 | 1.062 (0.184-6.143) | 0.947 |
| Intravenous antibiotic exposure in previous 90 Days | 2.029 (0.479-8.603) | 0.337 | 1.319 (0.226-7.713) | 0.759 |
| Trimethoprim-sulfamethoxazole exposure in previous 90 days | 2.167 (0.292-16.098) | 0.450 | 1.786 (0.218-14.613) | 0.589 |

aRR: adjusted risk ratio; CI: confidence interval; RR: risk ratio
